# Why anesthetic protocols do not travel intact: hidden curriculum and local reasoning in small-animal veterinary anesthesia

**DOI:** 10.3389/fvets.2026.1887524

**Published:** 2026-07-20

**Authors:** Shotaro Nagahama

**Affiliations:** 1 JAVA Incorporated Association, Tokyo, Japan; 2 Department of Veterinary Anesthesia, Veterinary Teaching Hospital, Faculty of Applied Biological Sciences, Gifu University, Gifu, Japan

**Keywords:** anesthetic protocols, clinical reasoning, hidden curriculum, patient-specific care, peri-anesthetic safety, protocol dependence, protocol transplantation, small-animal anesthesia

## Abstract

In small-animal veterinary anesthesia, protocols, standard combinations, and dose-based practical aids are highly visible parts of the educational and clinical landscape. This visibility is not inherently problematic, because protocol-like structures can improve efficiency and provide workable starting points. However, in dogs and cats, where care often allows repeated observation, incremental adjustment, and response-guided modification, quality depends heavily on patient-specific interpretation and management. In that setting, protocol dependence becomes especially problematic. This article advances three related claims. First, protocol dependence describes a drift in which a starting framework comes to stand in for patient-specific anesthetic thinking. Second, this problem can be interpreted through the lens of hidden curriculum: protocol-shaped educational units may remain more portable than the reasoning formally endorsed. Third, anesthetic protocols can be understood as local organizational instruments, so protocols borrowed from external experts do not necessarily travel intact across institutions, a problem this article refers to as protocol transplantation. The argument is not that protocols should be abandoned. Rather, protocols should be repositioned as scaffolds for local, patient-specific reasoning rather than treated as portable finished products. Improving small-animal anesthesia will require not only better protocols, but more explicit teaching of how anesthetic plans are interpreted, revised, and locally enacted.

## 1 Introduction

In small-animal veterinary anesthesia, the language of protocols, standard combinations, dose ranges, and practical drug-selection aids is unusually prominent. That prominence is not inherently problematic. Contemporary small-animal guidance documents explicitly emphasize individualized planning and patient-specific judgment, while also presenting anesthesia as a practical clinical framework for use before, during, and after anesthesia (1, 2).

This article does not simply restate that anesthesia should be individualized. That principle is already well established. Nor does it argue that veterinary anesthetists are unaware of the need for individualization. Rather, it argues that protocol-shaped educational units may remain more portable than the patient-specific reasoning that is formally endorsed. In this article, hidden curriculum refers to the implicit lessons learners acquire from what is repeatedly modeled, emphasized, and made practically usable, even when those lessons are not the formal instructional message (3, 4). Applied to small-animal anesthesia teaching, the concern is that compact protocol-shaped units may be learned as the most actionable part of the lesson, whereas patient-specific reasoning remains less portable. Against that background, this article advances three related claims. First, the central problem is protocol dependence: the drift by which protocols cease to serve as starting frameworks and begin to stand in for anesthetic thinking. Second, this problem can be interpreted through the lens of hidden curriculum, in which drug combinations and dose patterns may be transmitted more efficiently than the reasoning meant to govern their use. Third, anesthetic protocols can be understood as local organizational instruments, not free-floating drug lists. When copied across institutions, their form may travel more easily than their function. This article refers to that problem as protocol transplantation. The claim here is not that veterinary anesthesia literature has already defined protocols in these exact terms, but that local adaptation work in veterinary peri-anesthetic safety and the broader functions-versus-forms distinction offer a useful conceptual basis for doing so (5, 6).

The purpose of this article is therefore not to reject protocols, but to reposition them. In small-animal veterinary anesthesia, protocols should function as local scaffolds for patient-specific reasoning, not as portable finished products presumed to retain their value unchanged across settings.

## 2 What a protocol is—And is not

The term protocol is used broadly in veterinary anesthesia, but it does not describe a single thing. At least three distinct ideas are often compressed into the same word. The first is a predesigned framework: a structured starting plan intended to support safety, efficiency, and shared understanding. The second is a fixed recipe-like administration pattern: a recognizable combination of drugs and doses treated as something close to a ready-made answer. The third is a patient-specific anesthetic plan: a plan built for an individual patient, with explicit expectations about risks, endpoints, likely responses, and anticipated need for revision (1).

This distinction matters because criticism of “protocols” can otherwise become imprecise. Standardization is often useful and sometimes essential. The problem begins when a protocol ceases to be treated as a provisional framework and is instead treated as the anesthetic itself. At that point, fidelity to the remembered combination risks taking precedence over fidelity to the patient.

A further distinction is also important. In practice, a protocol is not merely a written drug list. It is enacted within a local clinical system involving particular personnel, monitoring capabilities, rescue pathways, and shared thresholds for intervention. For that reason, the same written protocol may represent a different practical intervention in different hospitals. This point is directly illustrated by veterinary peri-anesthetic checklist implementation work, while the broader functions-versus-forms literature is used here only as a conceptual aid for extending that insight to anesthetic protocols more generally (5, 6).

## 3 Why protocol culture became prominent in veterinary anesthesia

The prominence of protocol-oriented discourse in veterinary anesthesia likely reflects structural features of the profession. In many veterinary settings, anesthesia is not delivered exclusively by specialists, and practical teaching becomes attractive because it is easy to remember and easy to deploy. Drug combinations and dose ranges are concrete, portable, and reassuring.

That practicality is visible not only in peer-reviewed guidelines but also in the surrounding educational ecosystem. AAHA provides an intravenous induction sheet that presents drug combinations, dose ranges, cautions, comments, and dosing examples in a compact protocol-style format (7). FECAVA offers a case-based educational document explicitly titled Anesthetic Protocols: case studies, again packaging discussion around concrete protocol examples while also stating that protocols should be individualized (8). WSAVA publishes procedure-specific material for dog neutering that includes a section labeled “Protocol without controlled drugs” and then lists concrete drug combinations with mg/kg suggestions (9). Taken together, these are not isolated examples from a single organization but recurring examples of protocol-shaped educational packaging across multiple prominent veterinary bodies.

This does not mean that specialists are teaching incorrectly whenever they discuss concrete regimens. The point is narrower: what is easiest to transmit is not always what is most educationally complete. Learners may hear the caveat to individualize, yet retain most strongly the portable elements—the drug list, the sequence, and the mg/kg numbers. Hidden curriculum literature is relevant here because it provides a way to interpret how what is repeatedly modeled and most easily carried away may come to dominate what is only formally stated (3, 4). The resulting tension is central to this article: individualized reasoning is the formal principle, but protocol-shaped packages remain highly visible educational units.

## 4 Where protocols are appropriate, and where they become misleading

Any critique that ignores species and situational constraints would be incomplete. In some veterinary contexts, predefined dosing strategies are not merely convenient; they are bound up with the practical demands of the setting. Equine induction is a useful example, because behavioral, physical, and logistic constraints make front-loaded planning and consistency especially important (10).

The present concern is narrower. In dogs and cats, especially in routine small-animal practice, clinicians often have greater opportunity to observe responses, alter timing, adjust supportive care, and revise the plan in light of emerging physiology. In that setting, excessive protocol dependence is more problematic because it can blur the distinction between a starting plan and the anesthetic process itself. Recipe-like thinking becomes more likely to displace adaptive management. This is consistent with the individualized-plan emphasis of major small-animal guidance documents, but the point made here about “protocol dependence” is this article's conceptual extension rather than a phrase already established in those guidelines (1).

## 5 Small-animal anesthesia is not drug delivery but adaptive management

The central clinical claim of this article is straightforward: in small-animal anesthesia, success is not determined by whether a protocol was reproduced, but by whether the patient was appropriately managed. A protocol specifies intended pharmacologic input. An anesthetic, by contrast, is a time-dependent interaction between those inputs, the patient's physiology, the procedure's stimuli, and the clinician's ongoing interpretation. That way of framing anesthesia is compatible with major guidance documents that describe anesthesia as a plan spanning preanesthesia, induction, maintenance, and recovery, rather than as a single recipe (1).

For that reason, the same nominal induction protocol can lead to materially different clinical realities. One patient may remain easy to ventilate, whereas another may show airway instability. One may tolerate induction with minimal circulatory disturbance, whereas another may develop bradycardia, apnea, or exaggerated responses to stimulation. During maintenance, identical settings do not imply equivalent depth, respiratory consequences, or hemodynamic adequacy. What follows is not that anesthesia is chaotic, but that it cannot be reduced to a recipe. Small-animal anesthesia is therefore better viewed as adaptive patient management than as standardized drug delivery. The clinical examples in this paragraph are illustrative rather than tied to a single source; the underlying point is a conceptual one built on the general individualized-management stance of current guidelines.

## 6 The educational problem: What specialists are actually teaching

The educational tension becomes most apparent in specialist-led teaching. Specialists know that anesthesia must be individualized. The question is whether teaching consistently transmits that principle in a form that learners can actually carry into practice. There is reason to worry that it sometimes does not, not because specialists deny the importance of reasoning, but because teaching environments strongly favor transmission of what is concise, concrete, and portable. Drug combinations and dose ranges satisfy those conditions extremely well. Reasoning frameworks often do not. Hidden curriculum literature is useful here because it helps explain why learners may internalize what is repeatedly modeled and most easily exported, rather than what is only formally stated (3, 4). The application of that idea to small-animal anesthesia teaching is interpretive and is proposed here as a conceptual reading, not as something those papers directly studied.

This interpretation also aligns with broader veterinary education literature, which increasingly treats clinical reasoning as a competency requiring explicit teaching rather than passive absorption (11). It also resonates with patient-safety literature, where problems are not explained only as failures of individual vigilance but as products of systems, communication, workload, supervision, and organizational culture (12–15). Seen in that light, protocol dependence is not only a learner problem. It can also be viewed as a knowledge-packaging problem. The final step in that argument is again this article's synthesis rather than a direct claim from any one source.

## 7 What should specialists teach instead?

The alternative is not to stop teaching drugs. Drug knowledge remains indispensable. The proposed shift is one of educational hierarchy: drugs and doses should be taught within a reasoning framework, not as the framework itself. The center of instruction should move from “what I use” toward “what I am trying to achieve, what I expect to happen, what I am watching for, and what would make me change course.” Table 1 summarizes this proposed shift. That shift is consistent with the broader veterinary clinical-reasoning literature, which argues that reasoning benefits from explicit teaching and structured support (11).

**Table 1:** Shifts in teaching emphasis in small-animal veterinary anesthesia.

What learners may most readily take from specialist teaching	What specialist teaching should make more explicit
Drug combinations and preferred regimens	Clinical goals and intended endpoints
Fixed doses or favored mg/kg values	Starting ranges and adjustment logic
“What I use”	Why it is chosen, what is expected, and when to change course
Induction protocols as compact solutions	Induction as response-guided management
Successful protocol examples	Failure conditions and revision triggers
Pharmacology alone	Pharmacology plus monitoring interpretation
Reproducible regimens	Revisable management
Borrowed protocols	Locally adapted protocols
The expert's preferred protocol	The expert's reasoning process

The table does not argue that specialist teaching is inappropriate or that regimen-based teaching should be avoided. Rather, it suggests that drug combinations and dose suggestions should be embedded within a broader framework of goals, anticipated responses, monitoring interpretation, and local adaptation.

A reasoning-centered approach to anesthesia teaching would foreground questions such as the following: what is the main endpoint in this patient at this stage? Which adverse responses are most likely? Which monitor signals are genuinely decision-relevant? What findings would justify escalation, reduction, delay, supplementation, or revision of the plan? Which parts of the anesthetic are provisional rather than fixed? These are the questions that turn protocols back into scaffolds. This paragraph is prescriptive and should be read as this article's proposal, informed by clinical reasoning literature rather than directly derived from it.

A second shift is also needed. Specialists should teach not only how a protocol is constructed, but also where its usefulness comes from. That usefulness does not arise solely from the pharmacology written on the page. It also depends on organizational fit: who is available, what monitoring is reliable, how deterioration is recognized, what rescue measures are realistic, and how tasks are distributed. This is directly consistent with veterinary checklist implementation work, while the functions-versus-forms terminology is again used here as an interpretive extension rather than direct veterinary anesthesia evidence (5, 6). Specialists can still describe reasonable starting combinations, but the educational emphasis should fall on starting ranges, adjustment logic, anticipated trade-offs, organizational assumptions, and response-guided modification.

## 8 A reframed role for protocols in small-animal anesthesia

**Figure 1:**
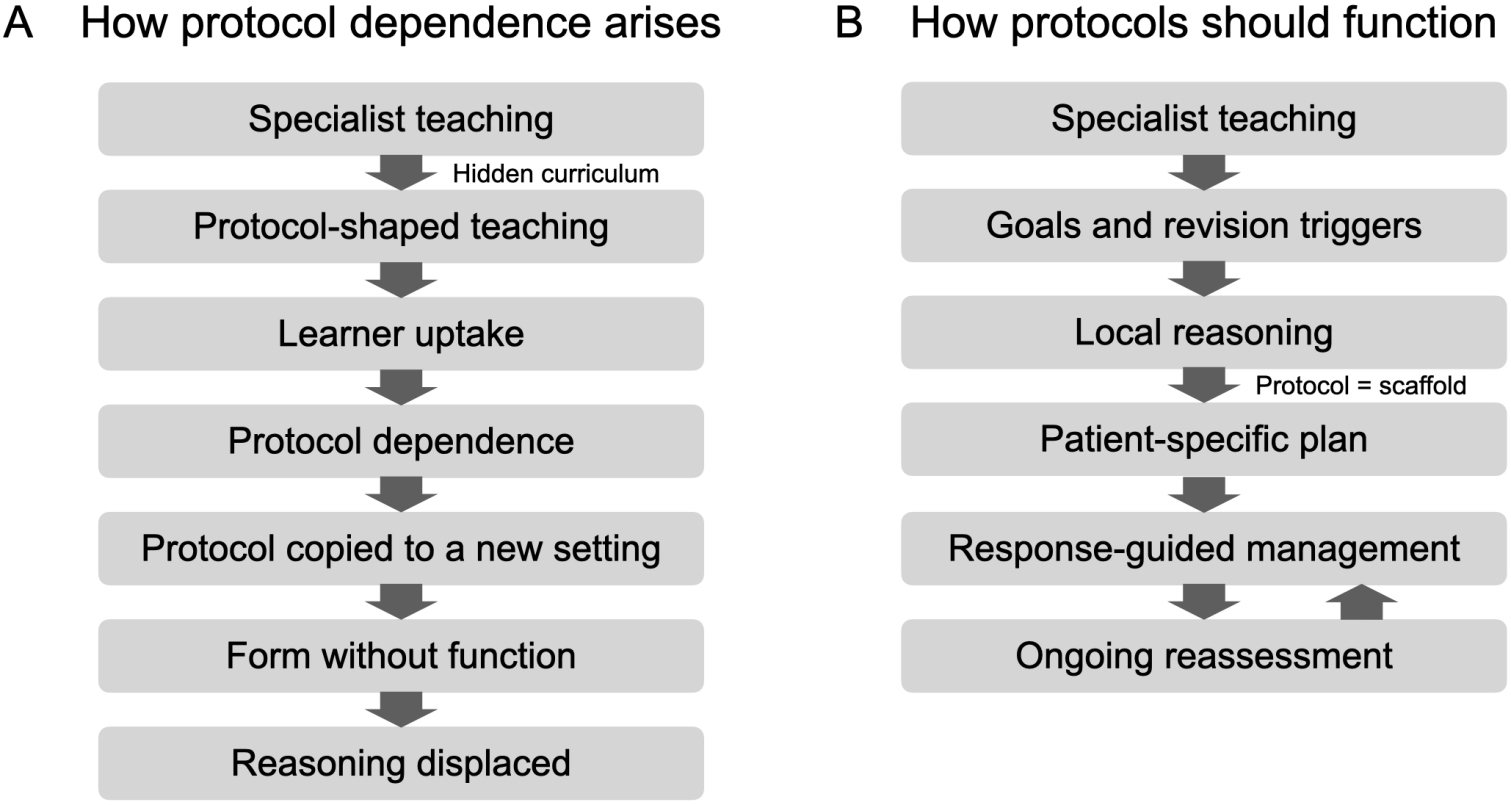
Conceptual pathways of protocol dependence and protocol use in small-animal veterinary anesthesia. **(A)** Protocol dependence may arise when protocol-shaped teaching is more portable than the reasoning formally endorsed, encouraging protocol copying across settings. **(B)** Protocols are better understood as scaffolds that support local reasoning, patient-specific planning, and response-guided management rather than as finished products to be transferred intact.

Protocols still have an important role in small-animal anesthesia. They can reduce omissions, promote team coordination, provide cognitive support under pressure, and offer learners a usable starting scaffold. Their value should not be minimized. The error lies not in protocol use, but in protocol elevation. Figure 1 summarizes the contrast between protocol dependence and the intended role of protocols as supports for local reasoning.

Reframed appropriately, protocols can be understood as local organizational scaffolds. Their apparent efficiency and safety are not free-floating properties of a drug list. They are achieved within a specific sociotechnical system: one with particular personnel, monitoring practices, communication routines, rescue capacity, and clinical goals. The portability of a protocol is therefore asymmetric. Drug names, doses, sequences, and timing can be copied relatively easily, but the less visible conditions that made the protocol work—who monitors, what equipment is available, how deterioration is recognized, and how rapidly the team can intervene—may not travel with it. The veterinary evidence directly supporting local adaptation comes from peri-anesthetic checklist implementation, whereas the language of functions and forms is borrowed here as a conceptual framework for thinking about why copied protocols may not behave identically across sites (5, 6). For that reason, a protocol borrowed from an external expert cannot be assumed to retain its usefulness when transplanted unchanged into another practice environment. This is the central implication of protocol transplantation: copying the visible form of a protocol does not ensure transfer of the function it served in its original setting. “Form travels more easily than function” is used here as a conceptual inference rather than a quoted conclusion from the cited articles.

This reframing also better aligns with the current small-animal guidance landscape. AAHA and ACVAA emphasize patient-specific planning and judgment, yet the same educational environment visibly circulates induction combination sheets, case-based protocol teaching materials, and procedure-specific protocol PDFs (1, 2, 7–9). The task is therefore not to abolish such aids, but to prevent their educational visibility from eclipsing the reasoning they are meant to support.

## 9 Conclusion

The central issue in small-animal veterinary anesthesia is not whether protocols exist, but what role they are allowed to play. Protocols are useful, often necessary, and sometimes deeply rational. Yet in dogs and cats, where repeated observation and response-guided adjustment are frequently possible, protocols should not function as substitutes for patient-specific anesthetic thinking.

This article has argued for three connected conceptual shifts. First, the relevant problem is protocol dependence, not protocol use. Second, protocol dependence can be interpreted not only as a learner problem but also as a knowledge-packaging problem, illuminated by hidden curriculum concepts. Third, anesthetic protocols can be fruitfully understood as local organizational instruments, whose practical function does not necessarily travel automatically when their written form is copied elsewhere.

Improvement will therefore require more than better protocol dissemination. It will require a more explicit effort to teach how anesthetic plans are interpreted, revised, and locally enacted. For that reason, the next step in improving small-animal anesthesia may not be the production of ever more refined protocols alone. It may be a more explicit commitment to teaching anesthesia as adaptive patient management: goal-directed, response-based, revisable, patient-specific, and locally enacted. Protocols should remain in veterinary anesthesia. They should simply be restored to their proper place—as scaffolds for thinking, not replacements for it.

## Data Availability

The original contributions presented in the study are included in the article/supplementary material, further inquiries can be directed to the corresponding author.
